# Correction to ‘Genomes OnLine Database (GOLD) v.8: overview and updates’

**DOI:** 10.1093/nar/gkae163

**Published:** 2024-02-28

*Nucleic Acids Research*, Volume 49, Issue D1, 8 January 2021, Pages D723–D733, https://doi.org/10.1093/nar/gkaa983

In the originally published online version of this manuscript, there were two co-authors missing from the author list: Tanja Woyke (https://orcid.org/0000-0002-9485-5637) and Emiley Eloe-Fadrosh (https://orcid.org/0000-0002-8162-1276).

The list should now read: ‘Supratim Mukherjee, Dimitri Stamatis, Jon Bertsch, Galina Ovchinnikova, Jagadish Chandrabose Sundaramurthi, Janey Lee, Mahathi Kandimalla, Tanja Woyke, Emiley A. Eloe-Fardosh, I-Min A. Chen, Nikos C. Kyrpides and T.B.K. Reddy’.

The emendation is outlined only in this correction notice to preserve the version of record.

